# Impact of norepinephrine on the relationship between pleth variability index and pulse pressure variations in ICU adult patients

**DOI:** 10.1186/cc10310

**Published:** 2011-07-12

**Authors:** Matthieu Biais, Vincent Cottenceau, Laurent Petit, Françoise Masson, Jean-François Cochard, François Sztark

**Affiliations:** 1 Emergency Department, Hôpital Pellegrin, Centre Hospitalier Universitaire de Bordeaux, Place Amélie Raba Léon, 33076 Bordeaux Cedex, France; 2 Université BORDEAUX Segalen, Rue Léo Saignat,33076 Bordeaux Cedex, France; 3 Service d'Anesthésie et de Réanimation 1, Hôpital Pellegrin, Centre Hospitalier, Universitaire de Bordeaux, Place Amélie Raba Léon, 33076 Bordeaux Cedex, France

## Abstract

**Introduction:**

Pleth Variability Index (PVI) is an automated and continuous calculation of respiratory variations in the perfusion index. PVI correlates well with respiratory variations in pulse pressure (ΔPP) and is able to predict fluid responsiveness in the operating room. ICU patients may receive vasopressive drugs, which modify vascular tone and could affect PVI assessment. We hypothesized that the correlation between PVI and ΔPP and the ability of PVI to identify patients with ΔPP > 13% is dependent on norepinephrine (NE) use.

**Methods:**

67 consecutive mechanically ventilated patients in the ICU were prospectively included. Three were excluded. The administration and dosage of NE, heart rate, mean arterial pressure, PVI and ΔPP were measured simultaneously.

**Results:**

In all patients, the correlation between PVI and ΔPP was weak (r^2 ^= 0.21; p = 0.001). 23 patients exhibited a ΔPP > 13%. A PVI > 11% was able to identify patients with a ΔPP > 13% with a sensitivity of 70% (95% confidence interval: 47%-87%) and a specificity of 71% (95% confidence interval: 54%-84%). The area under the curve was 0.80 ± 0.06. 35 patients (53%) received norepinephrine (NE(+)). In NE(+) patients, PVI and ΔPP were not correlated (r^2 ^= 0.04, p > 0.05) and a PVI > 10% was able to identify patients with a ΔPP > 13% with a sensitivity of 58% (95% confidence interval: 28%-85%) and a specificity of 61% (95% confidence interval:39%-80%). The area under the ROC (receiver operating characteristics) curve was 0.69 ± 0.01. In contrast, NE(-) patients exhibited a correlation between PVI and ΔPP (r^2 ^= 0.52; p < 0.001) and a PVI > 10% was able to identify patients with a ΔPP > 13% with a sensitivity of 100% (95% confidence interval: 71%-100%) and a specificity of 72% (95% confidence interval: 49%-90%). The area under the ROC curve was 0.93 ± 0.06 for NE(-) patients and was significantly higher than the area under the ROC curve for NE(+) patients (p = 0.02).

**Conclusions:**

Our results suggest that in mechanically ventilated adult patients, NE alters the correlation between PVI and ΔPP and the ability of PVI to predict ΔPP > 13% in ICU patients.

## Introduction

Fluid is administered to critically ill patients in order to increase cardiac preload and cardiac output (CO), yet studies have shown that about 50% of critically ill patients do not exhibit the desired effect [1,2]. None of the routinely used static variables of cardiac preload such as filling pressures (central venous pressure (CVP) and pulmonary artery occlusion pressure) reliably predict fluid responsiveness [1,3]. In contrast to static indices of preload, dynamic indices based on cardiopulmonary interactions and variations in left ventricular stroke volume are able to predict adequately the individual response to fluid loading [4-9] in specific settings (adult patients, tidal volume > 8 ml/kg, chest closed, heart rate/respiratory rate ratio > 3.6, absence of spontaneous breathing effort, arrhythmia, increase in intra-abdominal pressure or right ventricular dysfunction) [10-15]. However, these techniques are either invasive (respiratory variations in pulse pressure (ΔPP), stroke volume variations) with potential complications or are not continuous. The monitoring of the respiratory variations in pulse oximetry plethysmographic waveform amplitude (ΔPOP) has been proposed as a non-invasive method. Several studies have shown that ΔPOP correlates well with ΔPP and predicts fluid responsiveness in mechanically ventilated patients in the operating room and not in spontaneous breathing patients [16-19]. However, the agreement between ΔPOP and ΔPP and the ability of ΔPOP to predict fluid responsiveness in ICUs remains controversial [20,21]. The difficulty in obtaining an interpretable pulse oximetry plethysmographic waveform signal could explain this controversy. Furthermore, ΔPOP is not easily measured at the bedside and cannot be continuously monitored. Recently, the Pleth Variability Index (PVI) (Masimo Corp., Irvine, CA, USA), a novel algorithm allowing automated and continuous calculation of the respiratory variations in the perfusion index (PI), was proposed. The pulse oximeter waveform uses the two components of light absorption. PI is defined as the ratio between constant absorption and pulsatile absorption. It has been demonstrated that PVI correlates well with ΔPOP and ΔPP and that it can predict fluid responsiveness and the hemodynamic effect of positive end-expiratory pressure in the operating room and in the immediate postoperative period [22-24]. In ICUs, some patients receive vasopressive drugs such as norepinephrine (NE), which modify vascular tone. PVI measurements are influenced by vascular tone that may affect its pulsatile absorption component [21,25,26]. In this way, NE may affect PVI assessment. We hypothesized that NE use may impact (i) the correlation between PVI and ΔPP and (ii) the ability of PVI to identify patients with a ΔPP above 13% (threshold often used in clinical practice in order to indicate volume expansion) [7]. To test this hypothesis, PVI and ΔPP were simultaneously measured in consecutive patients receiving or not receiving NE. This hypothesis was tested only in adult patients because ΔPP fails in predicting fluid responsiveness in children because arterial compliance and chest wall-to-lung elastance ratio differ from adults [15].

## Materials and methods

### Patients

After obtaining approval from the local ethics committee (Comité de Protection des Personnes Sud-Ouest et Outre Mer III, Bordeaux, France; protocol no. DC 2009/34) and informed consent from the patient's next of kin, 67 consecutive mechanically ventilated patients from our ICU were prospectively included in the study.

Inclusion criteria were the following: patients mechanically ventilated without spontaneous breathing effort (identified by clinical examination and visual examination of respiratory curves), tidal volume of 8 ml/kg or above, absence of arrhythmia, heart rate/respiratory rate ratio above 3.6, Motor Activity Assessment Scale below 1 [27], Ramsay score above 5, absence of hypothermia or hyperthermia, left ventricular ejection fraction above 50%, absence of right ventricular dysfunction (attested by a peak systolic velocity of tricuspid annular motion < 0.15 m/s), and absence of increase in intra-abdominal pressure suspected by clinical context and examination. Patients were excluded if hemodynamic instability occurred (defined by a variation in heart rate or blood pressure of ≥10% over the 15-minute period before starting and during the protocol).

Sedation and analgesia were provided by continuous infusion of midazolam or propofol with sufentanil or morphine. All patients were previously equipped with an arterial catheter (115.090, 20 gauges, 8 cm, Vygon, Ecouen, France) connected and stored to a bedside monitor (Ultraview SL2900, Spacelabs Healthcare, Issaquah, Washington, USA). A personal computer was connected to the monitor in order to record arterial curves. A pulse oximeter probe (LNOP^® ^Adt, Masimo Corp., Irvine, CA, USA) was attached to the index finger of the controlateral hand and wrapped to prevent outside light from interfering with the signal. The pulse oximeter was connected to a Masimo Radical 7 monitor with PVI software (Masimo SET, Masimo Corp., Irvine, CA, USA).

### Measurements

#### Echocardiographic measurements

Doppler echocardiography was performed using a standard transthoracic probe (P4-2, Siemens Medical, Malvern, PA, USA) and a dedicated unit (Acuson CV-70, Siemens Medical System, Malvern, PA, USA). The stroke volume was calculated as the product of the aortic valve area by the velocity time integral of aortic blood flow (VTIAo). Using the parasternal long-axis view, the diameter of the aortic cusp was measured and the aortic valve area (π(diameter^2^)/4) was calculated. Using the apical five-chamber view, the VTIAo was computed from the area under the envelope of the pulsed-wave Doppler signal obtained at the level of the aortic annulus. The VTIAo value was averaged over five consecutive measurements. CO was calculated as the product of heart rate and stroke volume. Left ventricular ejection fraction (LVEF) was measured using the biplane Simpson's method from the apical two- and four-chamber views. Respiratory variations in VTIAo were measured: the maximum and minimum VTIAo values were identified for one minute and averaged to obtain VTIAomax and VTIAomin. The mean VTIAo (VTIAomean) was calculated as (VTIAomax - VTIAomin)/2. Respiratory variations in VTIAo was calculated as (VTIAomax - VTIAomin)/VTIAomean × 100. Peak systolic velocity of tricuspid annular motion, a right ventricular function parameter, was assessed by tissue Doppler echocardiography. Patients with a peak systolic velocity of tricuspid annular motion below 0.15 m/s were not included because it has been shown that ΔPP may prove inaccurate in this case [12].

#### Calculation of ΔPP

Arterial waveforms were recorded using a personal computer. The computer images were analyzed using Image J software (National Institutes of Health, Bethesda, MD, USA). Pulse pressure was defined as the difference between systolic and diastolic arterial blood pressure. Maximal (Pulse Pressure max) and minimal (Pulse Pressure min) values were determined over the same respiratory cycle. ΔPP was then calculated as: ΔPP = (Pulse Pressure max - Pulse Pressure min)/((Pulse Pressure max + Pulse Pressure min)/2), as previously described [7]. ΔPP was evaluated in triplicate over each of three consecutive respiratory cycles. The mean values of the three determinations were used for statistical analysis. ΔPP was analyzed offline by a physician (MB) blinded to PVI values.

#### Pleth Variability Index

For the measurement of oxygen saturation via pulse oximetry, red and infrared lights are utilized. A constant amount of light (DC) from the signal of the pulse oximeter is absorbed by the skin, other tissues, and nonpulsatile blood, while a variable amount of light (AC) is absorbed by the pulsating arterial inflow. To calculate PI, the infrared pulsatile signal is indexed against the nonpulsatile infrared signal and expressed as a percentage (PI = (AC/DC) × 100). The infrared signal is used because it is less affected by changes in arterial saturation than the red signal. PVI is a measure of the dynamic changes in the PI that occur during the respiratory cycle (PVI = (PImax-PImin)/Pimax). It is calculated by measuring changes in PI over a defined time interval where one or more complete respiratory cycle occurred. PVI is therefore displayed continuously on the monitor as a percentage.

#### Other measurements

Temperature, mean arterial pressure, and heart rate were also recorded.

### Study protocol

Immediately before and during the recording period, vasoactive drugs were not changed, and fluid expansion was not given. One set of simultaneous measurements was performed. PVI values were recorded by an observer blinded to ΔPP values. Measurements began after a stable PVI value (i.e., a value that remained unchanged or varied for a maximum of one point) was obtained for at least five minutes.

### Statistical analysis

Results were expressed as mean ± standard deviation unless stated otherwise. ΔPP and PVI were compared using Student's *t *test and linear correlation. Characteristics of patients receiving NE (NE(+)) or not (NE(-)) were compared using Student's *t *test. Data were divided into two groups according to the value of ΔPP (> 13% or ≤13%); this threshold has been shown to be predictive of fluid responsiveness in mechanically ventilated patients [7]. Receiver operating characteristic (ROC) curves were generated for PVI in all patients, in NE(+) patients and in NE(-) patients, varying the discriminating threshold of this parameter. Area under the ROC curves generated for NE(+) and NE(-) patients were compared using a z test. A *P *value of less than 0.05 was considered to be statistically significant. Statistical analysis was performed using Statview for Windows, version 5 (SAS Institute, Cary, NC, USA), Medcalc (software 11.5.1.0; Mariakerke, Belgium) and Sigmaplot 11.0 (Systat Software, Inc. San Jose, CA, USA).

## Results

### Global analysis

**Figure 1:**
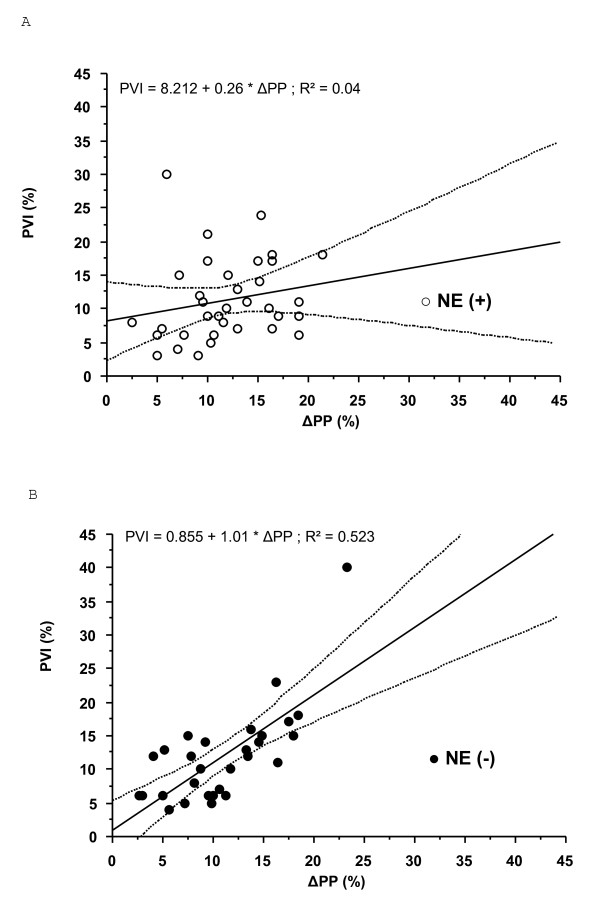
**Relation between Pleth Variabilty Index and respiratory-induced variations in pulse pressure in patients (a) receiving or (b) not receiving norepinephrine**. clear circle, patients receiving norepinephrine (NE(+)); filled circle, patients not receiving norepinephrine (NE(-)); ΔPP, respiratory-induced variations in pulse pressure; PVI, Pleth Variabilty Index.

Sixty seven patients were initially included. Three patients were excluded for hemodynamic instability during the protocol (*n *= 3). Patients were included 3.8 ± 1.7 days after admission to the ICU. The main characteristics of the 64 patients are shown in Table 1. In all patients, PVI and ΔPP were not significantly different (*P *> 0.05). The correlation coefficient between PVI and ΔPP was 0.21 (*P *= 0.001; Figure 1). In all patients, respiratory variations in VTIAo correlated moderately with PVI (r^2 ^= 0.19, *P *= 0.0003). Twenty-three patients exhibited a ΔPP above 13%. In all patients, a PVI threshold value above 11% was able to discriminate between ΔPP above 13% and ΔPP of 13% and below with a sensitivity of 70% (95% confidence interval: 47% to 87%) and a specificity of 71% (95% confidence interval: 54% to 84%). Positive and negative predictive values and positive and negative likelihood ratio are shown in Table 2. Area under the ROC curve for PVI to predict ΔPP above 13% was 0.80 ± 0.06.

**Table 1:** Main characteristics of patients

Characteristics	*n *= 64
Age (years)	45 ± 19
Height (cm)	171 ± 9
Weight (kg)	73 ± 13
Body mass index (kg/m^2^)	25 ± 4
Gender, F/M (n)	22/42
SAPS II	35 ± 14
Aetiologies of ICU admission:	
- Polytraumatism	26
- Brain injury	21
- Postoperative:	
- Orthopaedic	7
- Abdominal	10
Temperature (°C)	37.0 ± 0.4
Norepinephrine (μg/kg/min)	0.20 ± 0.27
Pulsatility index	2.7 ± 2.6
Inspiratory O_2 _fraction (%)	40 ± 11
Tidal volume (ml/kg)	8.6 ± 0.7
Respiratory rate (/min)	15 ± 2
Positive end expiratory pressure (cmH_2_O)	4 ± 2
Heart rate/respiratory rate (ratio)	5.4 ± 1.3

Data are expressed as number or mean ± SD.

F, female; M, male; SAPS, Simplified Acute Physiology Score; SD, standard deviation.

**Table 2:** Impact of norepinephrine on the ability of PVI to discriminate between ΔPP > 13% and ΔPP ≤13%

	PVI threshold	Sensitivity (95% confidence interval)	Specificity (95% confidence interval)	+PV	-PV	+LR	-LR
**All patients**	11%	70% (47%-87%)	71% (55%-84%)	57%	81%	2.38	0.43
**NE(+)**	10%	59% (28%-85%)	61% (39%-80%)	44%	74%	1.49	0.68
**NE(-)**	10%	100% (71%-100%)	72% (47%-90%)	69%	100%	3.60	0.00

ΔPP, respiratory-induced variations in pulse pressure; +LR, positive likelihood ratio; -LR, negative likelihood ratio; NE(+), patients receiving norepinephrine; NE(-), patients not receiving norepinephrine; +PV, positive predictive values; -PV, negative predictive values; PVI, Pleth Variabilty Index.

### Impact of norepinephrine

Thirty five patients (53%) received NE since 3.6 ± 1.6 days (mean dosage = 0.36 ± 0.27 μg/kg/min). None of them received dobutamine, epinephrine or dopamine. Hemodynamic data, temperature, inspiratory oxygen fraction, tidal volume, respiratory rate, and heart rate were not different in NE(+) and NE(-) patients (Table 3).

**Table 3:** Main characteristics of patients receiving (NE(+)) or not receiving (NE(-)) norepinephrine

	NE(+) (*n *= 35)	NE(-) (*n *= 29)	*P*
Temperature (°C)	37.0 ± 0.4	37.0 ± 0.4	ns
HR (/min)	82 ± 19	88 ± 24	ns
MAP (mmHg)	83 ± 11	84 ± 16	ns
LVEF (%)	67 ± 9	64 ± 12	ns
CO (l/min)	4.6 ± 1.3	5.4 ± 2.0	0.04
PI	2.7 ± 2.7	2.6 ± 2.4	ns
ΔPP (%)	12 ± 5	11 ± 5	ns
PVI (%)	11 ± 6	12 ± 7	ns

Data are expressed as mean ± standard deviation. ΔPP, respiratory-induced variations in pulse pressure; CO, cardiac output; HR, heart rate; LVEF, left ventricular fraction ejection; MAP, mean arterial pressure; NE(+), patients receiving norepinephrine; NE(-), patients not receiving norepinephrine; PI, Pulsatility Index; PVI, Pleth Variabilty Index.

**Figure 2:**
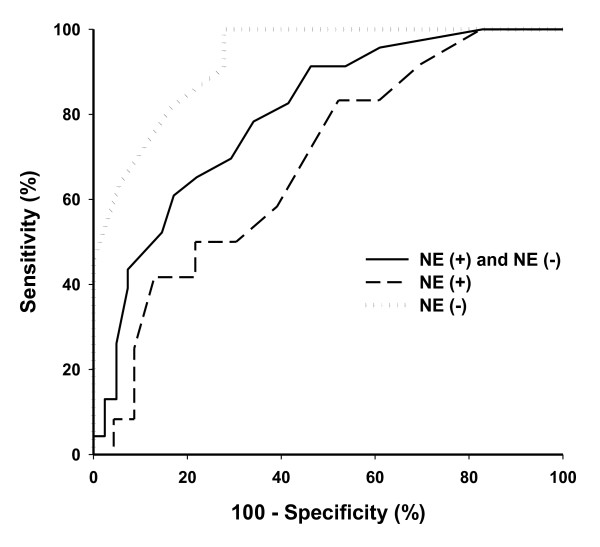
**Receiver operating characteristic curves showing the ability of PVI to discriminate patients with ΔPP above 13% in all patients and in patients receiving NE(+) or not receiving NE(-) norepinephrine**. ΔPP, respiratory-induced variations in pulse pressure; PVI, Pleth Variabilty Index.

In NE(+) patients, PVI did not correlate with ΔPP (r^2 ^= 0.04, *P *> 0.05; Figure 1) or with respiratory variations in VTIAo (r^2 ^= 0.031, *P *> 0.05). Twelve patients (24%) exhibited a ΔPP above 13%. A PVI threshold value above 10% was able to discriminate between ΔPP above 13% and ΔPP of 13% or below with a sensitivity of 58% (95% confidence interval: 28% to 85%) and a specificity of 61% (95% confidence interval: 39% to 80%). Positive and negative predictive values and positive and negative likelihood ratio are shown in Table 2. Area under the curve for PVI to predict ΔPP above 13% in NE(+) patients was 0.69 ± 0.01 (Figure 2). The difference between ΔPP and PVI was not correlated with NE dosage (*P *> 0.05).

In NE(-) patients, PVI correlated with ΔPP (r^2 ^= 0.52, *P *< 0.001; Figure 1) and with respiratory variations in VTIAO (r^2 ^= 0.53, *P *< 0.0001). Eleven patients (38%) exhibited a ΔPP above 13%. A PVI threshold value above 10% was able to discriminate between ΔPP above 13% and ΔPP of 13% or below with a sensitivity of 100% (95% confidence interval: 71% to 100%) and a specificity of 72% (95% confidence interval: 49% to 90%). Positive and negative predictive values and positive and negative likelihood ratio are shown in Table 2. Area under the curve for PVI to predict ΔPP above 13% was 0.93 ± 0.06 (Figure 2).

The area under the ROC curves for NE(+) and NE(-) patients were significantly different (*P *= 0.02).

### Impact of pulsatility index

PI values were not correlated with NE dosage (*P *> 0.05). The difference between PVI and ΔPP was not correlated with PI values (*P *> 0.05 in all patients, both in NE(+) patients and in NE(-) patients).

## Discussion

Our data suggest that in the condition of the study, the relation between PVI and ΔPP in ICU patients is weak and that the ability of PVI to predict a ΔPP above 13% are negatively influenced by NE use. Further studies are needed to investigate the ability of PVI to predict fluid responsiveness in specific settings.

Mechanical ventilation induces cyclic changes in intrathoracic and transpulmonary pressures that transiently affect left ventricular preload, resulting in cyclic changes in stroke volume in preload-dependent, but not in preload-independent, patients [28,29]. These cyclic changes in stroke volume can be evaluated by cyclic changes in arterial pulse pressure. Several studies have shown that ΔPP is able to predict fluid responsiveness in patients in the operating theatre and in ICUs [4,30]. Even if most patients admitted to ICUs are instrumented with an intra-arterial catheter, it is well known that percutaneous arterial catheterization is associated with rare but serious complications (thrombosis, infections, pseudoaneurysm, hematoma, bleeding) [31-35]. Therefore, a non-invasive approach may be of value.

ΔPOP has been proposed as an alternative to ΔPP. In the operating room, it has been shown that ΔPOP correlates well with ΔPP and can predict fluid responsiveness [16,17]. In ICUs, however, the results are controversial. Although some studies have found a strong correlation between ΔPOP and ΔPP and have shown that ΔPOP can be a good indicator of fluid responsiveness [20,36], Landsverk et al. showed a poor agreement between ΔPOP and ΔPP [21]. This technique is not yet available in clinical practice because plethysmographic waveform processing and filtering requires specific tools and software that are not widely available. PVI has been proposed for the automated and continuous calculation of the respiratory variations in the pulse oximeter waveform amplitude [22]. It can also predict fluid responsiveness in the operating room [23].

In the present study, the correlation between PVI and ΔPP is weak in ICU patients. Our results are not in accordance with those of Loupec et al. [37]. Several reasons may explain these differences. First, the population studied by Loupec et al. is different from our patients. They included a majority of surgical and septic patients. In contrast, we included a majority of trauma patients. In patients with brain injury, NE is administered in order to increase cerebral perfusion pressure, whereas peripheral vasomotor tone is hardly affected. In contrast, in septic patients, vasoplegia induces an alteration in microvascular perfusion and NE is administered in order to improve or restore vasomotor tone. The amplitude of the pulse oximetry plethysmographic waveform is influenced by changes in vascular tone from all tissue compartments present in the fingertip, and vasoconstriction narrows the amplitude of the waveform. Thus, patients with brain injury who require NE had potentially a different vasomotor tone than patients with septic shock under NE and this may affect PVI in a different manner. Unfortunately, we did not explore skin microcirculation (e.g. using laser Doppler flowmetry) and we cannot make firm conclusions on this hypothesis. Second, in the present study the mean duration of NE infusion before starting protocol was rather long (3.6 ± 1.6 days) and all patients did not receive NE. This may impact the quality of the signal. Third, we did not perform fluid challenge or passive leg raising tests. Finally, we excluded patients with right ventricular dysfunction.

Mechanical ventilation induces cyclic changes in stroke volume. These variations may be measured ideally at the level of the heart (e.g. respiratory variations in VTIAo) or may be evaluated peripherally using surrogates (ΔPP, pulse contour analysis). As PVI and ΔPP are measured peripherally, NE infusion may alter the relation between these surrogates and the respiratory variations in stroke volume. In this way, the lack of correlation between ΔPP and PVI in NE(+) patients could be due to issues in PVI or ΔPP. However, the relation between PVI and respiratory variations in VTIAo was also altered in NE(+) patients. Thus, the lack of relation between PVI and ΔPP in NE(+) patients seems to be due to issues in PVI, and not in ΔPP.

Several mechanisms are known to interfere with ΔPOP and PVI calculations and could explain our results. PI depends on vasomotor tone, which may affect the pulsatile absorption component [25,26]. For this reason, signal quality, body temperature, vasoactive drug infusion, level of sedation, presence of nociceptive input, and spontaneous movements may have an impact. Even if vasomotor tone is constant over a single respiratory cycle and does not alter the analysis of the relative change in PI induced by mechanical ventilation, it may be that vasoconstriction induced by NE narrows the amplitude of the pulse oximeter waveform and alters the PI analysis. We did not observe a dose-effect relation (the difference between ΔPP and PVI was not correlated with NE dosage). A recent study suggested that PI values may influence the ability of PVI to predict fluid responsiveness [38]. In our study, we did not find any relation between PI values and NE dosage or between PI values and the difference between PVI and ΔPP values. Pulse oximeter waveform may be influenced by outside light absorption and the pulse oximeter may have to be wrapped in order to prevent outside light from interfering with the signal. Furthermore, the site for measuring the effect of ventilation on pulse oximeter waveform (ear, finger, and forehead) is of major importance [39]. In order to avoid artefacts, all these parameters have been taken into account during the procedure.

Our study has some limitations. First, we focused on the relation between PVI and ΔPP and did not perform fluid challenge. We chose 13% as a cut-off value for ΔPP because this was the first value to be reported and because most of the studies focusing on this topic found similar values even in patients receiving NE [7,40]. However, there is no firm data supporting any discriminant universal ΔPP threshold in clinical settings, especially in patients needing tight fluid titration. Second, as dynamic indices such as ΔPP or PVI are known to have some limitations, we made sure we were testing situations in which these indices are interpretable: no spontaneous breathing activity, no arrhythmia, tidal volume above 8 ml/kg, absence of right heart failure and heart rate/respiratory rate above 3.6 [10-12]. Finally, we did not measure intra-abdominal pressure in all patients whereas it is known that clinical context and evaluation may cause problems when diagnosing intra-abdominal hypertension [41,42].

## Conclusions

In the conditions of the study, our results suggest that: the correlation between PVI and ΔPP is weak; NE modifies the correlation between PVI and ΔPP; and NE alters the ability of PVI to predict ΔPP above 13% in ICU patients.

## Key messages

• The relation between PVI and ΔPP is weak in ICU patients.

• The relation between PVI and ΔPP is negatively influenced by NE use.

• The ability of PVI to predict a ΔPP above 13% is negatively influenced by NE use.

## Abbreviations

ΔPOP: respiratory variations in pulse oximetry plethysmographic waveform amplitude; ΔPP: respiratory induced variations in pulse pressure; CO: cardiac output; CVP: central venous pressure; LVEF: left ventricular ejection fraction; NE: norepinephrine; PI: perfusion index; PVI: pleth variability index; ROC: receiver operating characteristics; VTIAo: velocity time integral of aortic blood flow.

## Competing interests

The authors declare that they have no competing interests. The manufacturers (Masimo Corp, Irvine, CA, USA) provided the material free of charge.

## Authors' contributions

MB conceived and designed the study. MB, VC, LP, and JFC collected the data. MB and FM performed the statistical analysis and drafted the manuscript. MB and FS wrote the paper. All authors read and approved the final manuscript
